# Hsa_circ_0085576 promotes clear cell renal cell carcinoma tumorigenesis and metastasis through the miR-498/YAP1 axis

**DOI:** 10.18632/aging.103300

**Published:** 2020-06-15

**Authors:** Guanghua Liu, Jingmin Zhou, Yuanlin Piao, Xin Zhao, Yuzhi Zuo, Zhigang Ji

**Affiliations:** 1 Department of Urology, Peking Union Medical College Hospital, Chinese Academy of Medical Science, Beijing 100730, P.R. China; 2 Department of Traditional Chinese Medicine, Peking Union Medical College Hospital, Chinese Academy of Medical Science, Beijing 100730, P.R. China

**Keywords:** clear cell renal cell carcinoma, has-hsa_circ_0085576, microRNA-498, yes-associated protein 1

## Abstract

There is emerging evidence that circular RNAs (circRNAs) act as important regulators in various cancers. It is less clear, however, what role circRNA plays in the tumorigenesis and metastasis of clear cell renal cell carcinoma (ccRCC). In this study, using bioinformatics analysis and a series of experimental analysis, we characterized a novel circRNA, hsa_circ_0085576 was up-regulated in ccRCC tissues and cell lines. High hsa_circ_0085576 expression was significantly correlated with tumor size, clinical stage, and metastasis status and poorer survival. Knockdown of hsa_circ_0085576 notably inhibited cell proliferation, migration, invasion, whereas enhanced cell apoptosis of ccRCC cells, *in vitro*. In contrast, overexpression of hsa_circ_0085576 had the opposite effects. Moreover, hsa_circ_0085576 silencing significantly suppressed tumor growth and metastasis, whereas overexpression of hsa_circ_0085576 had the opposite effects, *in vivo*, Our results further showed that hsa_circ_0085576 acted as a competitive endogenous RNAs to interact with microRNA-498, to attenuate its repressive effect on target gene Yes-associated protein 1 (YAP1). Finally, functional studies revealed that inhibition of hsa_circ_0085576 suppressed cell growth and metastasis by regulating miR-498/YAP1 signaling, in ccRCC cells. Based on these findings, hsa_circ_0085576 may represent a valuable prognostic biomarker and a potential therapeutic target to curb the tumorigenesis and metastasis of ccRCC.

## INTRODUCTION

Renal cell carcinoma (RCC) is one of the most common tumors originated from renal tubular epithelial cells with a high mortality rate worldwide [1]. Clear cell renal cell carcinoma (ccRCC) is the most common subtype of RCC and accounts for 80% of all kidney cancers [2]. Early surgical resection remains the recommended treatment option for most ccRCC. Although surgery may be curative for early-stage ccRCC patients, deaths from RCC have not declined on account of recurrence and metastasis [3]. At present, plenty of genes have been found to participate in the pathogenesis of ccRCC, such as Prostaglandin E2 receptor 4 [4], Sirtuin 5 [5] and G3BP1 [6]. At the same time, some noncoding RNAs have also been identified to involve in the biological processes of this disease, such as microRNA-765 [7], long noncoding RNA (lncRNA) TP73-AS1 [8] and circRNA ZNF609 [9]. However, the exact pathogenesis of ccRCC still needs to be further clarified. Thus, a better understanding of the molecular mechanisms underlying aggressive ccRCC and exploring novel therapeutic strategies for ccRCC treatment is needed.

Circular RNAs (circRNAs) are a newly appreciated class of RNAs found across phyla that are generated most commonly from back-splicing of protein-coding exons. It is more stable and not easily degraded by exonuclease when comparable to linear RNA [10]. CircRNAs are present in eukaryotic cells and considered as competitive endogenous RNAs (ceRNAs) via “sponging” microRNAs (miRNAs), working as transcription factors. Besides, abnormal circRNAs expression could lead to alteration of gene products that contributing to tumor biology including cell proliferation, apoptosis, and metastasis [11]. For example, circRNA hsa-circ-0072309 exhibited inhibitory roles on cell survival by targeting miR-100 by blocking the PI3K/AKT and mTOR cascades in the Caki-1 and ACHN cell lines [12]. CircRNA cRAPGEF5 plays a role in suppressing RCC via the miR-27a-3p/TXNIP pathway and served as a promising prognostic biomarker and therapeutic target for RCC treatment [13], and circ-AKT3 suppresses ccRCC metastasis by enforcing E-cadherin expression through competitively binding miR-296-3p [14]. However, the mechanistic and functional characterization of circRNAs in ccRCC is still largely unknown.

In this study, we focused on the upregulated circRNAs based on the data from circRNAs microarray of ccRCC tissues, and identified that hsa_circ_0085576, which has not been reported before, was significantly upregulated in ccRCC tissues and cell lines. The present study demonstrated that elevated hsa_circ_0085576 was positively associated with the clinical pathological stage and may serve as a candidate prognostic biomarker. Mechanically, our data revealed that hsa_circ_0085576 could directly sponge miR-498 to upregulate YAP1 expression and consequently promote the growth and metastasis of ccRCC. Hsa_circ_0085576 may serve as an oncogene to promote ccRCC metastasis and be applied to a novel therapeutic target.

## RESULTS

### CircRNAs profiling in ccRCC tissues and has-hsa_circ_0085576 characterization

To determine the circRNAs profiling in ccRCC tissues, we used the microarray gene profiling data of ccRCC GSE100186 and GSE137836. GSE100186 consists of 3 normal, 3 RCC samples and GSE137836 consists of 3 primary tumor tissues and 3 humans metastatic RCC samples. As shown in Supplementary Figure 1A–1D, 3541 circRNAs were dysregulated in the GSE100186 dataset (1919 upregulated and 1622 downregulated); and 981 circRNAs were differentially expressed in the GSE137836 dataset (455 upregulated and 526 downregulated). Further overlap analysis showed that 13 circRNAs were consistently up-regulated and 2 circRNAs were down-regulated in above two datasets (Supplementary Figure 1E, 1F, Supplementary Table 2). According to the GO categories in the ccRCC tissues, the top 3 enriched biological processes corresponding to the mRNAs up-regulated in the pilocarpine model compared with those in the control were RNA polymerase II transcription factor binding, calcium ion-regulated exocytosis of neurotransmitter, and transcription cofactor binding (Supplementary Figure 1G). For the KEGG pathway, the most enriched KEGG pathway corresponding to the circRNAs up-regulated in the GSE100186 and GSE137836 dataset was the YAP1 signaling pathway (Supplementary Figure 1H).

**Figure 1:**
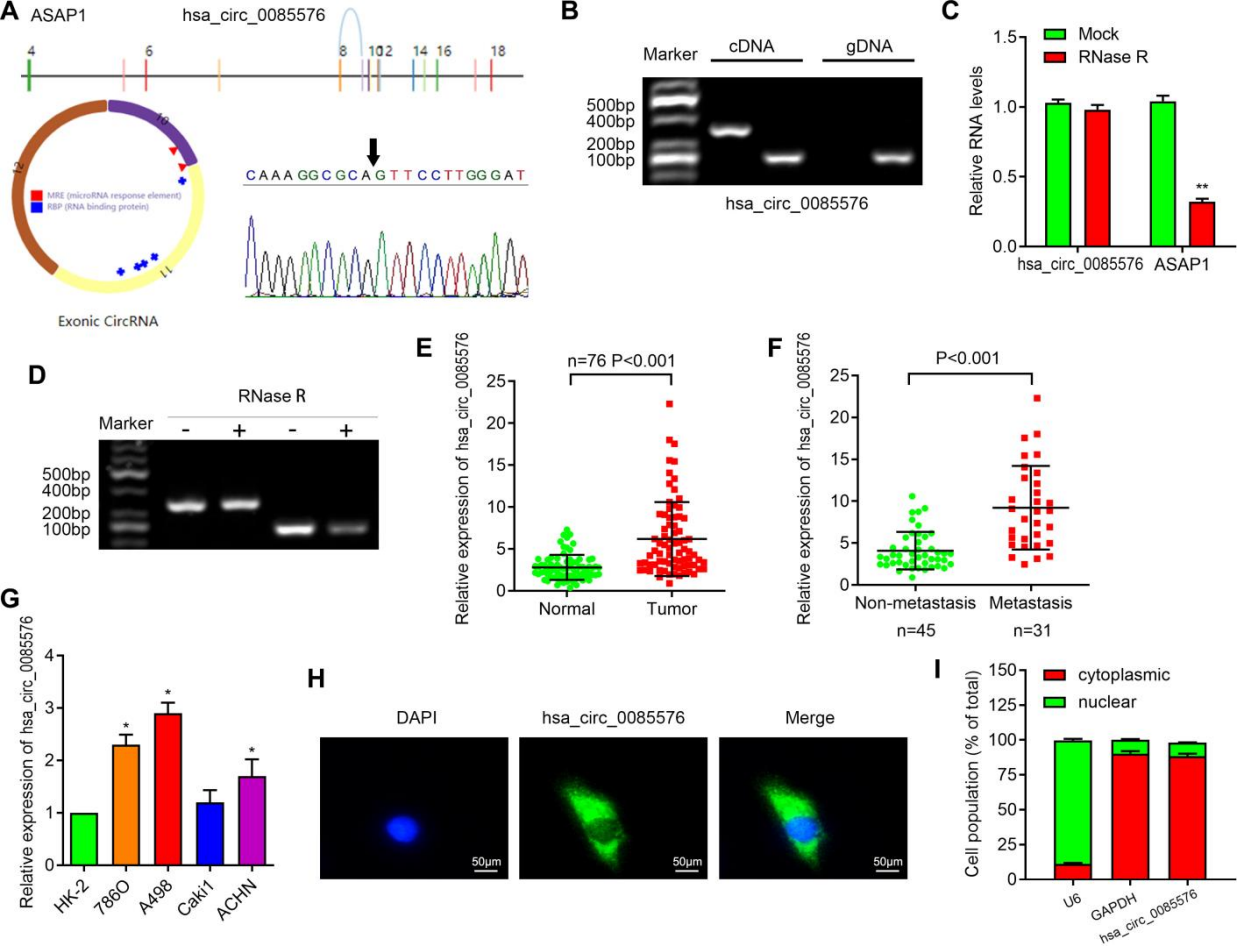
**Characterization of hsa_circ_0085576 in ccRCC cells.** (**A**) Schematic diagram of the genomic location and splicing pattern of has-hsa_circ_0085576; the specific primers of 0085576 were validated by Sanger sequencing. (**B**) The existence of hsa_circ_0085576 was validated by RT-PCR in ccRCC tumor tissue samples. (**C**, **D**) RT-qPCR was used to determine the abundance of hsa_circ_0085576 and linear ASAP1 mRNA in A498 cells treated with RNase R. (**E**) The levels of hsa_circ_0085576 in 40 paired ccRCC and matched adjacent normal tissues were examined by RT-qPCR. (**F**) The levels of hsa_circ_0085576 in 45 non-metastasis and 31 metastasis ccRCC were examined by RT-qPCR. (**G**) The expression of hsa_circ_0085576 in cell lines HK-2, 786-O, A498, Caki1, and ACHN was detected by RT-qPCR. (**H**) The cellular distribution of hsa_circ_0085576 in A498 cells was analyzed by fluorescence in situ hybridization (FISH). Green indicates hsa_circ_0085576 and blue indicates nuclei. (**I**) The cellular distribution of hsa_circ_0085576 was analyzed by cellular RNA fractionation assays. U6 and GAPDH were used as nuclear and cytoplasmic positive controls, respectively. * P<0.05 vs. HK-2; ** P<0.05 vs. mock.

We then focused on the most significantly upregulated circRNA has-hsa_circ_0085576, which is located on chromosome 8, and consists of 3 exons (exons 10–12) from its host gene ASAP1 (ArfGAP with SH3 domain, ankyrin repeat and PH domain 1) genome. Sanger sequencing then confirmed the head-to-tail splicing of hsa_circ_0085576 with the expected size (Figure 1A). Subsequently, we used cDNA and genomic DNA from selected ccRCC tissue as template to amplify hsa_circ_0085576 from cDNA using only divergent primers, while no amplification product was observed from genomic DNA (Figure 1B). We then applied RNase R, a processive 3′ to 5’ exoribonuclease, to digest total RNAs and found that compared with linear ASAP1, hsa_circ_0085576 was significantly resistant to RNase R, implying that hsa_circ_0085576 is circular (Figure 1C, 1D). Afterward, RT-qPCR analysis found that hsa_circ_0085576 was significantly upregulated in the ccRCC tissues when compared with adjacent normal tissues (Figure 1E), and further upregulated in the invasive ccRCC tissues when compared with those in the non-invasive tumor tissues (Figure 1F). The expression of endogenous hsa_circ_0085576 was the highest in A498 cells, moderate in 786O and ACHN cells, and was the lowest in Caki1 cells (Figure 1G). The FISH analysis and cell subcellular fraction assay against hsa_circ_0085576 showed that it was mostly located in the cytoplasm and rarely expressed in the nucleus (Figure 1H, 1I).

### Upregulated hsa_circ_0085576 is correlated with unfavorable prognosis in ccRCC

**Figure 2:**
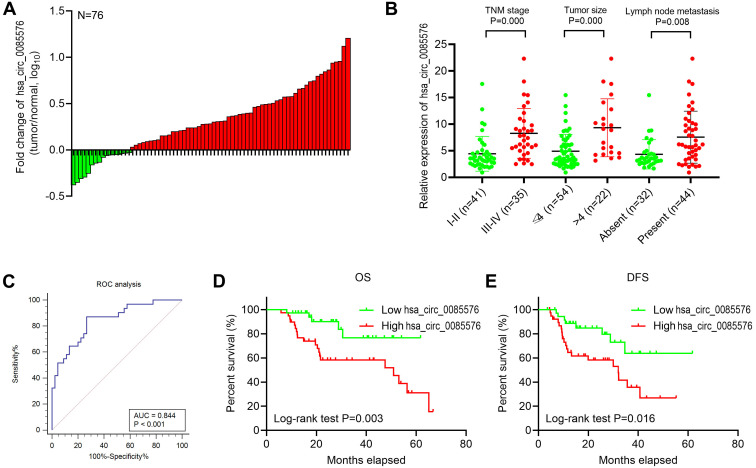
**Hsa_circ_0085576 is related to the clinicopathological characteristics of ccRCC patients.** (**A**) Fold changes of hsa_circ_0085576 in 40 paired ccRCC tissues. (**B**) High hsa_circ_0085576 expression was associated with advanced TNM stage, tumor size and lymph node metastasis. (**C**) Receiver operating characteristic (ROC) analysis was used to determine the cut-off value of hsa_circ_0085576 in 45 non-metastasis and 31 metastasis ccRCC patients. Cut-off value=4.447. (**D**) Association of hsa_circ_0085576 expression with OS analysis of 76 ccRCC patients. (**E**) Association of hsa_circ_0085576 expression with DFS analysis of 76 ccRCC patients.

We further explore the association between the hsa_circ_0085576 expression level and clinical significance. The fold change of hsa_circ_0085576 in the tumor tissues and adjacent normal ones were shown in Figure 2A. Primarily, we found that that hsa_circ_0085576 was significantly higher in patients with advanced stage, large tumor size, or lymph node metastasis (Figure 2B). We then assigned the above indicated 76 patients into two groups according to the levels of hsa_circ_0085576. The average level of hsa_circ_0085576 was defined as the cut-off value (4.447) by using ROC analysis (Figure 2C). hsa_circ_0085576 was positively correlated with the clinical stage, tumor stage and distant metastasis. Nevertheless, hsa_circ_0085576 levels were not associated with other clinical characteristics, containing age (P=0.531) and gender (P=0.517) (Table 1). Additionally, multivariate cox regression analysis revealed that clinical stage, tumor stage, distant metastasis and high hsa_circ_0085576 expression are independent predictors of OS in patients with ccRCC (Table 2). Kaplan-Meier survival analysis then showed poorer overall survival (OS) and disease-free survival (DFS) of ccRCC patients with high hsa_circ_0085576 levels (Figure 2D, 2E).

**Table 1:** Correlation between circ0085576 expression and clinicopathological features of 76 patients with clear cell renal cell carcinoma.

**Clinical characteristics**	**circ0085576 expression^a^**		***P* value^b^**
	**Low**	**High**	
Age(years)			0.532
<60	12	9	
≥60	27	28	
Gender			0.517
Male	26	22	
Female	13	15	
Clinical stage			0.006
I-II	27	14	
III-IV	12	23	
Tumor stage			0.012
T1+ T2	24	12	
T3+ T4	15	25	
Distant metastasis			0.000
M0	29	10	
M1	10	27	

a: circ0085576 high group (n=37) and low group (n=39) is defined due to tis median value of 86 patients. b: Pearson χ2 test was used to derive *P-*values.

**Table 2:** Univariate and multivariate analyses of clinicopathological factors for overall survival of patients with clear cell renal cell carcinoma.

**Risk factors**	**Univariate analysis**		**Multivariate analysis**	
	**HR (95%CI)**	**P-value**	**HR (95%CI)**	**P-value**
Age(years)	1.034(0.352-2.324)	0.914		
Gender	0.942(0.565-2.905)	0.465		
Clinical stage	1.789(1.019-4.856)	0.004	1.664(1.017-4.186)	0.028
Tumor stage	2.523(1.075-4.093)	0.007	2.081(1.128-3.597)	0.010
Distant metastasis	3.066(1.324-7.784)	0.000	2.866(1.324-6.784)	0.002
circ0085576 expression	1.476(1.098-5.889)	0.015	1.372(1.077-5.151)	0.032

### Hsa_circ_0085576 promotes ccRCC cell growth and metastasis, in vitro

**Figure 3:**
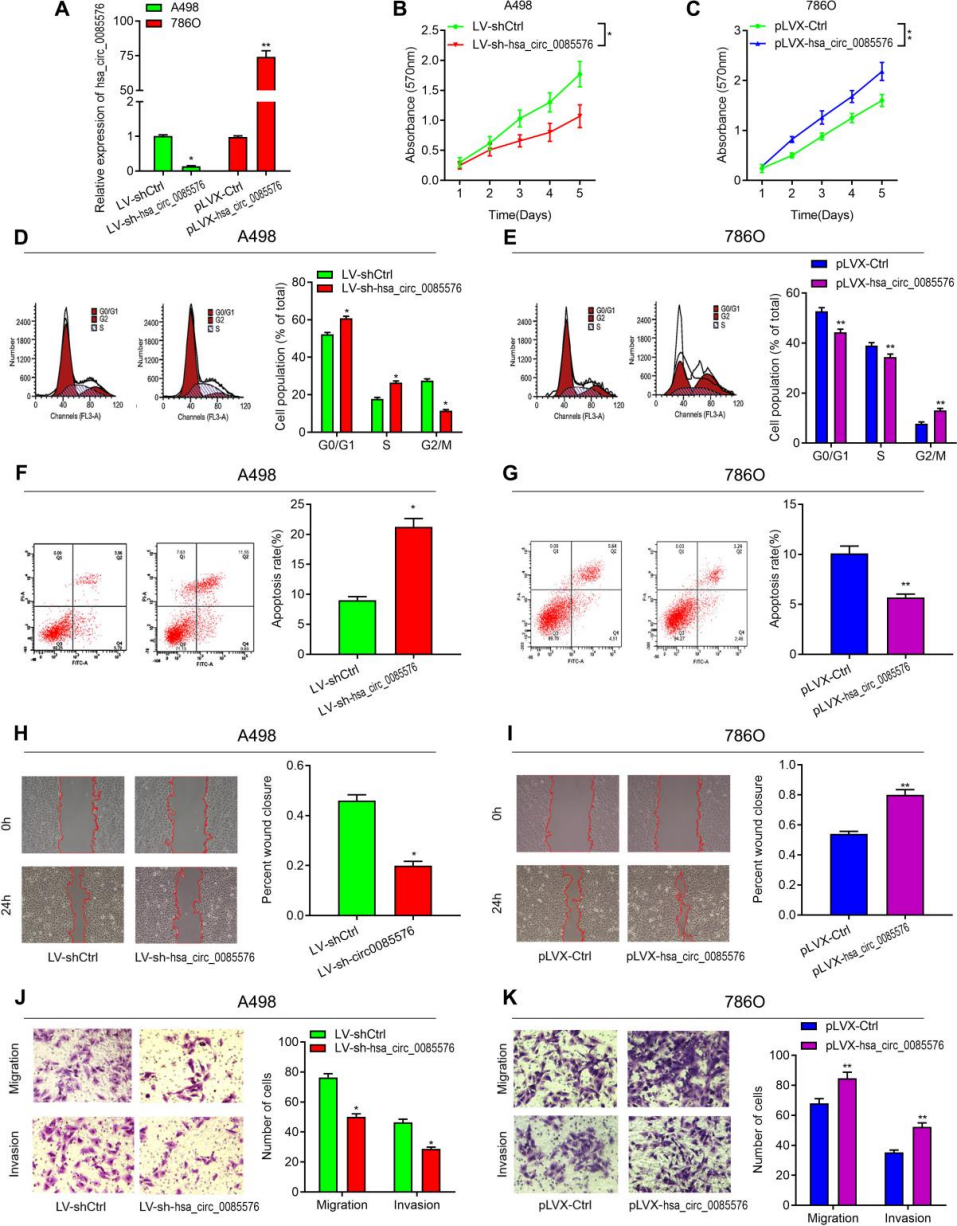
**Hsa_circ_0085576 promotes cell proliferation, cell cycle, migration and invasion, and inhibits cell apoptosis, in vitro.** (**A**) RT-qPCR analysis of hsa_circ_0085576 levels in A498 cells transfected with LV-sh-hsa_circ_0085576 or LV-shCtrl and in 786O cells transfected with pLVX-hsa_circ_0085576 or pLVK-Ctrl. (**B**, **C**) A498 or 786O cell proliferation after the expression of hsa_circ_0085576 was down-regulated or up-regulated, respectively, as assessed by CCK-8 assay. (**D**, **E**) A498 cells transfected with LV-sh-hsa_circ_0085576 or LV-shCtrl and 786O cells transfected with pLVX-hsa_circ_0085576 or pLVK-Ctrl were stained by propidium iodide and analyzed using flow cytometry. (**F**, **G**) flow cytometry was used to determine the apoptotic rates of A498/LV-sh-circ0085567 or 786O/pLVX-circ0085567. (**H**, **I**) the wound-healing assay showed A498 and 786O cell mobility after the expression of hsa_circ_0085576 was down-regulated or up-regulated, respectively. (**J**, **K**) Transwell assay showed A498 and 786O cell migration and invasion after the expression of hsa_circ_0085576 was down-regulated or up-regulated, respectively. * P<0.05 vs. LV-sh-Ctrl; ** P<0.05 vs. pLVX-Ctrl.

**Figure 4:**
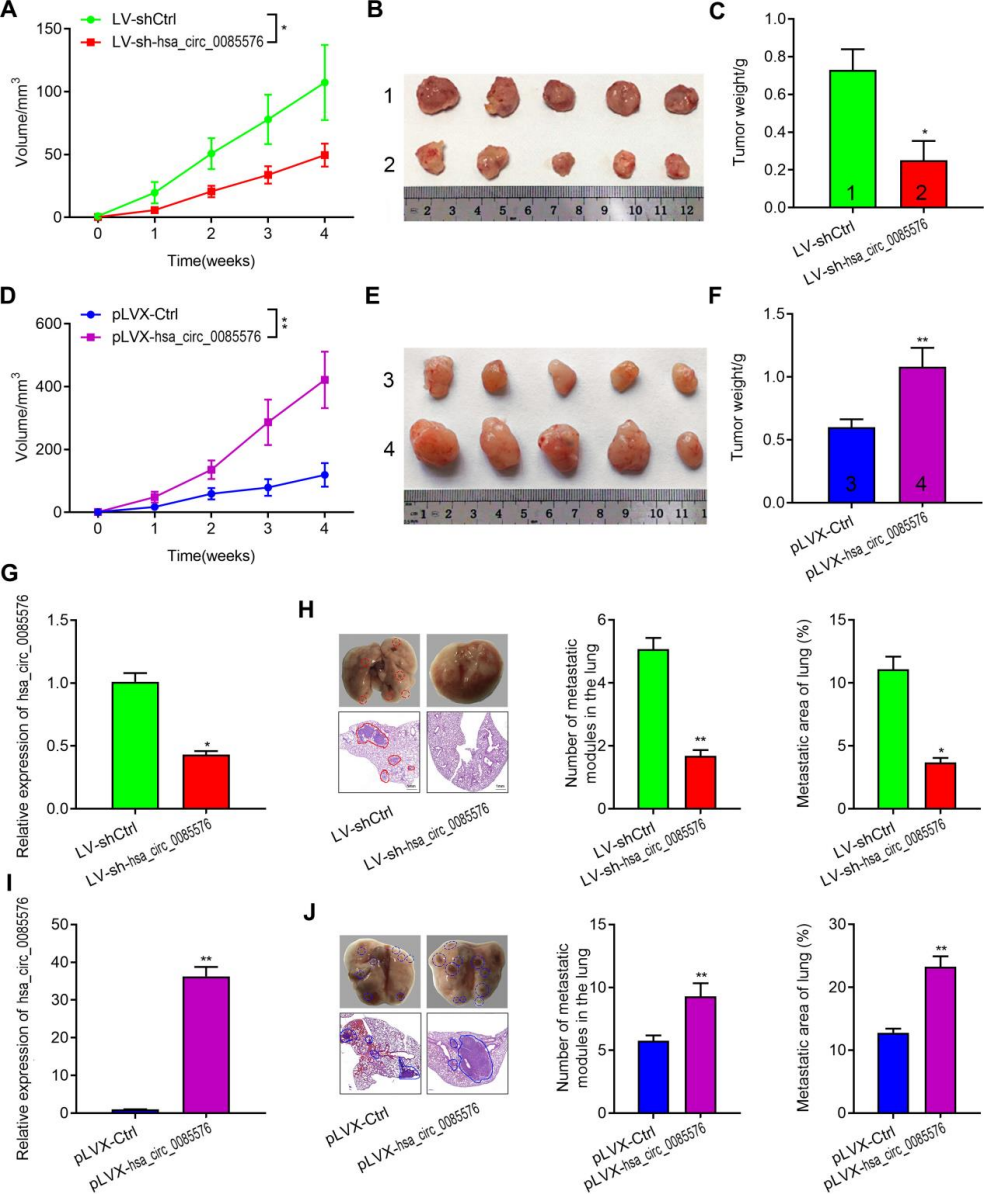
**Hsa_circ_0085576 promotes cell growth and metastasis of ccRCC in vivo.** (**A**–**F**) A, Tumor volumes of A498/LV-sh-hsa_circ_0085576 were measured every week for 4 weeks. B, Images of subcutaneous xenograft tumors of A498/LV-sh- hsa_circ_0085576 cells. C, the final tumor weight of A498/LV-sh-hsa_circ_0085576 cells was shown. D, Tumor volumes of 786O/pLVX-hsa_circ_0085576 cells were measured every week for 4 weeks. E, Images of subcutaneous xenograft tumors of 786O/pLVX-hsa_circ_0085576 cells. F, the final tumor weight of 786O/pLVX-circ0085567 cells was shown. (**G**, **H**) the expression of hsa_circ_0085576 was detected by RT-qPCR analysis in tumors with A498/LV-sh-hsa_circ_0085576 or 786O/pLVX-hsa_circ_0085576. (**I**, **J**) Stably transfected A498 cells with LV-sh-hsa_circ_0085576 or 786O cells with pLVX-hsa_circ_0085576 were injected into the vein of BALB/c nude mice for 4 weeks. Representative images of lungs (metastatic nodules were indicated by arrows) and H&E staining of lung metastatic lesions was shown. The number of metastatic nodules and metastasis areas in the lungs of BALB/c nude mice is quantified for each group (n=6). * P<0.05 vs. LV-sh-Ctrl; ** P<0.05 vs. pLVX-Ctrl.

To investigate the biological functions of hsa_circ_0085576 in ccRCC, LV-sh-hsa_circ_0085576 and pLVX-hsa_circ_0085576 vectors were constructed, and the efficiency of infection was verified by RT-qPCR (Figure 3A). CCK-8 assay showed that the down-regulation of hsa_circ_0085576 significantly inhibited cell proliferation of A498 cells (Figure 3B), whereas overexpression of hsa_circ_0085576 increased that of 786O cells (Figure 4C). Cell cycle analysis suggested that down-regulation of hsa_circ_0085576 increased G1/S phase arrest (Figure 3D), and overexpression of hsa_circ_0085576 promoted the G1/S phase transition (Figure 3E). For the analysis of cell apoptosis, inhibition of hsa_circ_0085576 promoted apoptosis of A498 cells (Figure 3F), whereas enhanced GINS4 expression inhibited apoptosis of 786O cells (Figure 3G). Besides, wound healing assay and transwell migration and invasion assays showed that down-regulation of hsa_circ_0085576 notably suppressed the ability of mobility, migration and invasion (Figure 3H, 3J), while up-regulation of hsa_circ_0085576 facilitated the ability of mobility, migration and invasion (Figure 3I, 3K).

### Hsa_circ_0085576 promotes ccRCC cell growth and metastasis, *in vivo*

We then confirmed the function of hsa_circ_0085576 in cell growth and metastasis, *in vivo*. The tumor growth model showed that hsa_circ_0085576 knockdown notably inhibited tumor growth whereas overexpression of hsa_circ_0085576 facilitated tumor growth (Figure 4A, 4D). Meanwhile, the sizes and weights of tumors in hsa_circ_0085576 knockdown group were markedly lower than those in the control group (Figure 4B, 4C), and the tumors in the hsa_circ_0085576 overexpressing group tumors were larger than its corresponding control group (Figure 4E, 4F). Additionally, RT-qPCR was used to confirm that the expression of hsa_circ_0085576 was lower in the LV-sh-circ0085567 group when compared with the LV-shCtrl group (Figure 4G), while higher expression of hsa_circ_0085576 was shown in the pLVX-hsa_circ_0085576 group when compared with the pLVX-Ctrl group (Figure 4I). The lung metastasis model then showed that the number of macroscopic and microscopic metastatic nodules formed in the lungs was significantly lower in hsa_circ_0085576 knockdown group (Figure 4H), whereas the numbers were higher in hsa_circ_0085576 overexpressing group (Figure 4J).

### The YAP1 signaling pathway may be a functional downstream pathway of hsa_circ_0085576

**Figure 5:**
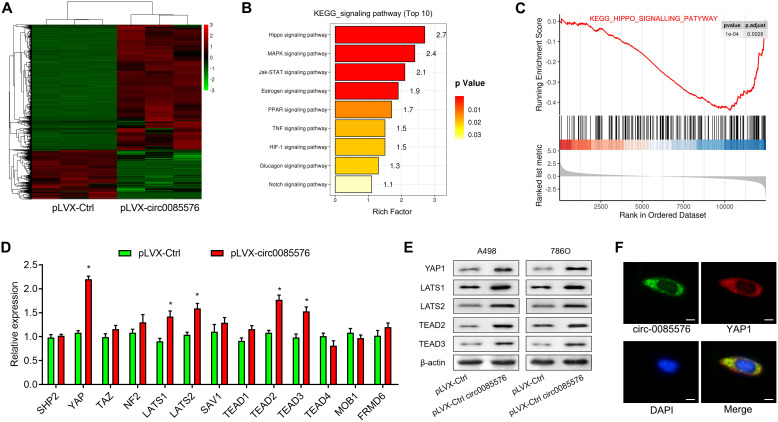
**Hsa_circ_0085576 promotes ccRCC progression through the YAP1 signaling pathway.** (**A**) Differentially expressed genes between the pLVX-Ctrl and pLVX-hsa_circ_0085576 group were shown in the heatmap. Red indicates upregulated; green indicates downregulated. (**B**) All enriched pathways in Kyoto Encyclopedia of Genes and Genomes (KEGG) analysis with statistical significance. (**C**) Gene set enrichment analysis (GSEA) showed hsa_circ_0085576 has a significant correlation with Hippo signaling pathway. (**D**) The mRNA levels of the related genes in the Hippo signaling pathways in 786O cells treated with pLVX-hsa_circ_0085576. (**E**) Western blot analysis of Hippo signaling pathway (YAP1, LATS1, LATS2, TEAD2 and TEAD3) in A498/LV-sh-hsa_circ_0085576, and 786O/pLVX-hsa_circ_0085576. (**F**) The double FISH showed that hsa_circ_0085576 and YAP1 were relatively co-localized in the cytoplasm of A498 cells. # P<0.05 vs. pLVX-Ctrl group.

RNA sequencing was then performed to explore the potential functional signaling pathways related to hsa_circ_0085576 in 786O/pLVX-hsa_circ_0085576 cells. Overexpression of hsa_circ_0085576 leads to the 148 up-regulated genes and 54 down-regulated genes (Figure 5A). The KEGG pathway analysis revealed that hsa_circ_0085576 affected many signaling pathways, including the Hippo, MAPK, and Jak-STAT signaling pathways (Figure 5B). Since the Hippo signaling pathway showed the highest rich factor and plays an important role in cell growth and metastasis during tumorigenesis [15], we investigated the involvement of Hippo signaling pathway during the regulatory role of hsa_circ_0085576 in ccRCC. In addition, GSEA analysis found the gene set of KEGG Hippo signaling pathway was significantly enriched in pLVX- hsa_circ_0085576 group compared to pLVX-Ctrl group (Figure 5C). The analysis of related genes in the Hippo signaling pathways demonstrated the YAP1 axis showed the most differential expression in the pathway (Figure 5D). Subsequent western blot analysis confirmed that overexpression of hsa_circ_0085576 activated YAP1 signaling pathway, as evidenced by increased YAP1, LATS1, LATS2, TEAD2 and TEAD3 (Figure 5E). Moreover, immunofluorescence confirmed the colocalization of hsa_circ_0085576 and YAP1 in 786O/pLVX-hsa_circ_0085576 cells (Figure 5F).

### Hsa_circ_0085576 sponges miR-498 in ccRCC

**Figure 6:**
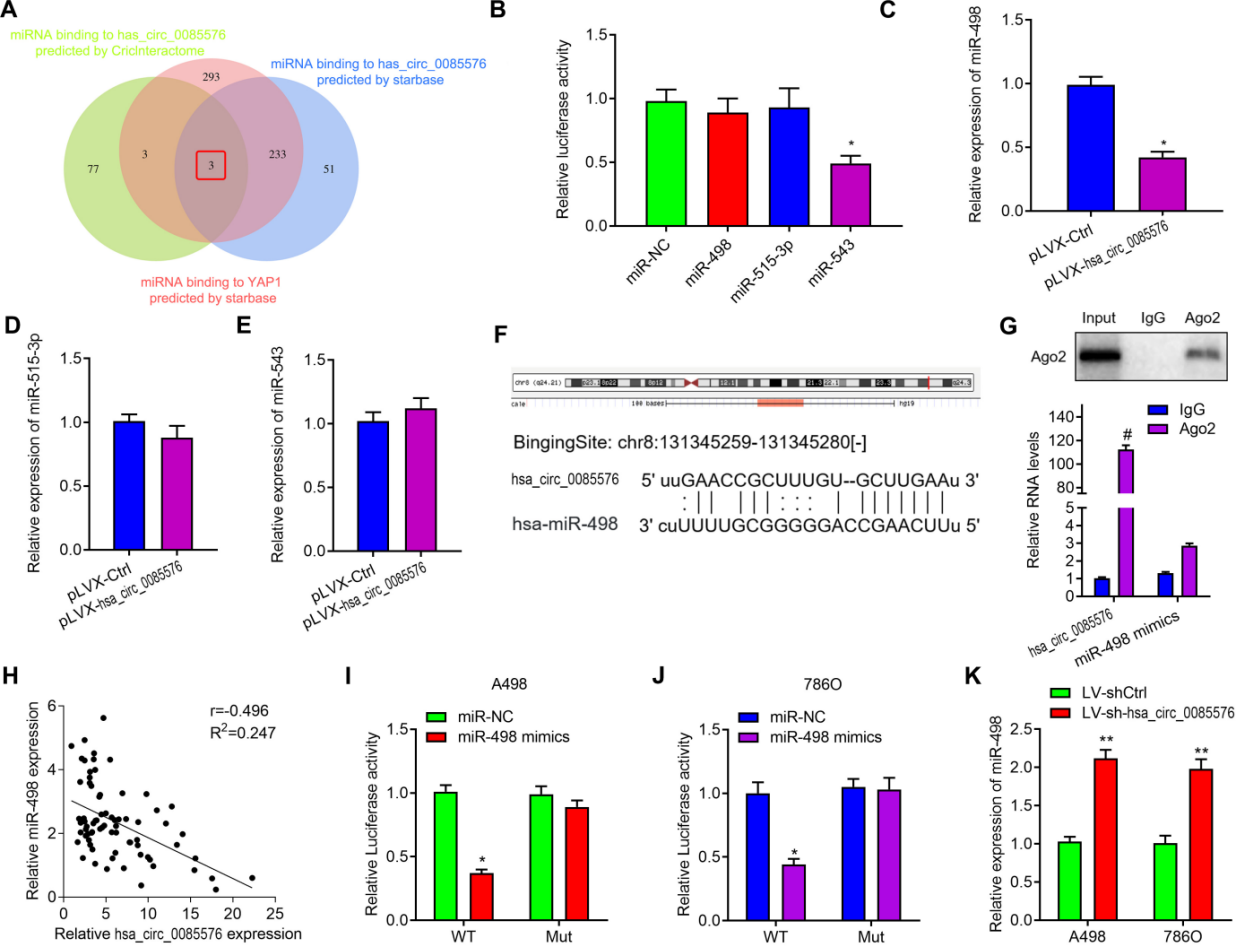
**Hsa_circ_0085576 may function as a sponge for miR-498.** (**A**) Venn diagram showing the mutual putative target genes of hsa_circ_0085576 and YAP1 predicted by StarBase and CircInteractome. (**B**) Dual-luciferase assays showing the luciferase activity of the pmiR-RB-hsa_circ_0085576 vector in 293T cells co-transfected with indicated miRNA mimics. (**C**–**E**) Relative expression of miR-498, miR-515-3p and miR-543 in 786O cell infected with pLVX-Ctrl or pLVX-hsa_circ_0085576. (**F**) A schematic drawing showing the putative miR-498 binding sites with respect to hsa_circ_0085576. (**G**) RIP experiments were performed using an anti-AGO2 antibody in 786O cells. (**H**) Hsa_circ_0085576 was inversely correlated with the expression of miR-498 (r = −0.405, P< 0.001) ccRCC tissues. (**I**, **J**) Luciferase activity assays were performed in A498 and 786O cells co-transfected with reporter plasmid (or the corresponding mutant reporter) and the indicated miRNAs. (**K**) Relative expression of miR-498 in A498 cells infected with LV-shCtrl or LV-sh-hsa_circ_0085576. * P<0.05 vs. miR-NC; ** P<0.05 vs. LV-shCtrl; # P<0.05 vs. pLVX-Ctrl.

Given the evidence that circRNAs exert its role as a ceNRA by interacting with miRNAs, StarBase 2.0 [16] and CircInteractome [17] were used to predict the underlying miRNA binding genes of hsa_circ_0085576 and YAP1. Intriguing, only three miRNAs were over-lapped by using above two web tools (Figure 6A). We then performed dual-luciferase assays in 293T cells to validate the regulation of hsa_circ_0085576 on above miRNAs and the results showed that only miR-498 reduced the luciferase reporter activity, indicating that hsa_circ_0085576 could bind to miR-498 (Figure 6B). As expected, overexpression of hsa_circ_0085576 did not influence the expression of miR-543 and miR-515-3p, but decreased the expression of miR-498 in 786O cells (Figure 6C–6E). Figure 6F showed the putative position of the miR-498 target sites in the 3’ UTR of hsa_circ_0085576 mRNA. RIP assay showed the enrichment of hsa_circ_0085576 and miR-498 could be combined with Ago2 protein in 786O cells (Figure 6G). subsequent correlation analysis found circ0085576 was negatively correlated with miR-498 expression in ccRCC tissues (Figure 6H). Co-transfection of miR-498 mimics and the WT vector significantly reduced luciferase activity, but this phenomenon was not observed for transfection of the Mut luciferase reporter in both A498 (Figure 6I) and 786O (Figure 6J) cells. Conversely, we found knockdown of hsa_circ_0085576 in A498 and 786O cells notably increased miR-498 expression (Figure 6K).

### YAP1 is a downstream target of miR-498 in ccRCC

**Figure 7:**
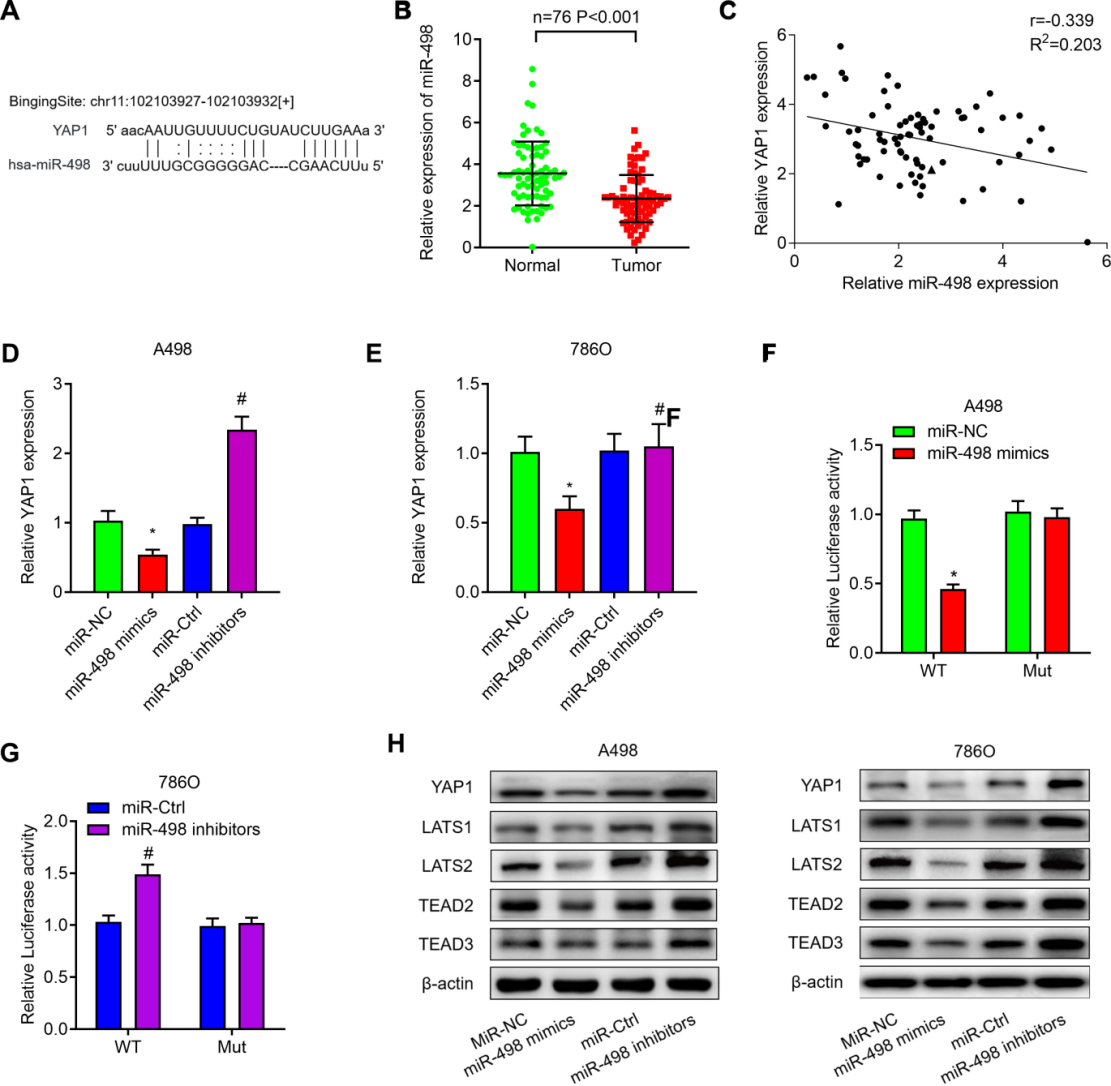
**MiR-498 suppresses YAP1 expression by directly binding to the YAP1 mRNA 3’UTR.** (**A**) The putative binding sites of miR-498 in YAP1 3’-UTR region were predicted. (**B**) Relative expression of miR-498 in 76 paired ccRCC tissues. (**C**) miR-498 was inversely correlated with the expression of miR-498 (r = −0.405, P< 0.001) ccRCC tissues. (**D**, **E**) Relative expression of YAP1 mRNA in A498 and 786O cells transfected with miR-NC, miR-498 mimics, miR-Ctrl or miR-498 inibitors. (**F**, **G**) A498 and 786O cells were transfected with YAP1 wild type reporter or mutant reporter constructs together with miR-498 mimics or inhibitors, and the luciferase activity was analyzed. (**H**) Western blot analysis of Hippo signaling pathway (YAP1, LATS1, LATS2, TEAD2 and TEAD3) in A498 and 786O cells transfected with miR-NC, miR-498 mimics, miR-Ctrl or miR-498 inibitors. * P<0.05 vs. miR-NC group; ** P<0.05 vs. miR-Ctrl group.

We then investigated whether miR-498 regulated YAP1 expression in ccRCC. The potential binding sites of miR-498 on YAP1 were predicted by TargetScan and the potential binding sites were listed in Figure 7A. The expression of miR-498 was decreased in ccRCC tissues when compared with the matched normal tissues (Figure 7B). Correlation analysis found miR-498 was negatively correlated with YAP1 mRNA expression in ccRCC tissues (Figure 7C). In addition, we found that miR-498 mimics could significantly decrease the expression of YAP1 mRNA, whereas miR-498 inhibitors could increase YAP1 expression in both A498 (Figure 7D) and 786O cells (Figure 7E). Luciferase reporter assay was performed to verify that miR-498 directly interacted with YAP1. MiR-498 mimics significantly inhibited luciferase activity of wild type reporter for YAP1, however, miR-498 did not inhibit the luciferase activity of reporter vector containing the mutant binding sites of YAP1 (Figure 7F). Meanwhile, miR-498 inhibitors enhanced luciferase activity of wild type reporter for YAP1, but not for mutant one (Figure 7G). Subsequently, western blot analysis demonstrated that overexpression of miR-498 markedly suppressed YAP1 signaling pathway, whereas knockdown of miR-498 activated YAP1 signaling, as evidenced by the changes of YAP1, LATS1, LATS2, TEAD2 and TEAD3 protein expression (Figure 7H).

### Hsa_circ_0085576 promotes ccRCC progression through the miR-498/YAP1 axis

**Figure 8:**
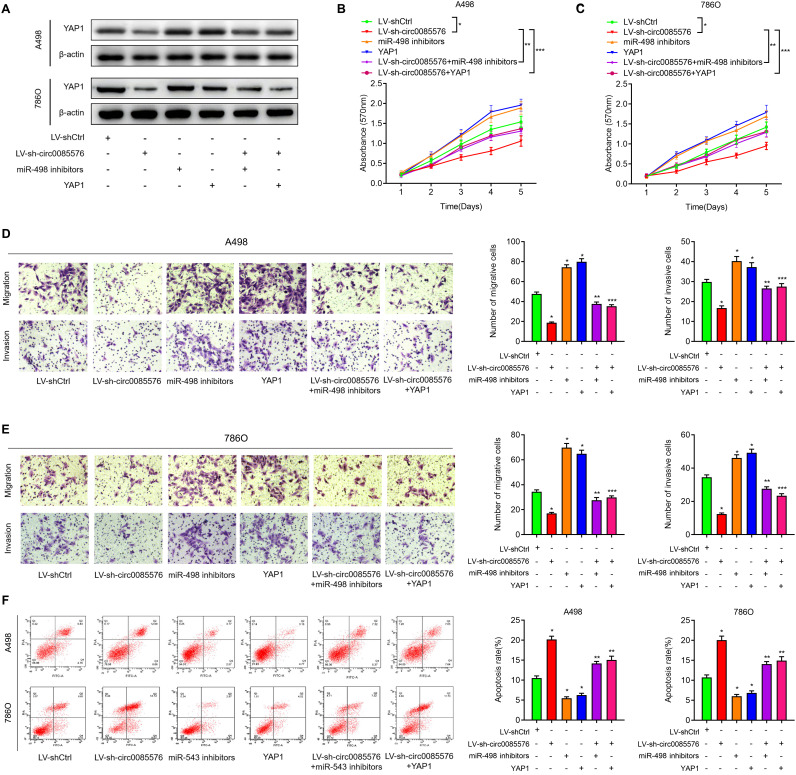
**The oncogenic hsa_circ_0085576/miR-498/YAP1 axis in ccRCC cells.** (**A**) Western blot analysis of YAP1 expression levels in A498 cells and 786O cells transfected with LV-shCtrl, LV-sh-hsa_circ_0085576 miR-498 inhibitors or YAP1 plasmid. (**B**, **C**) CCK-8 assay was used to detect the cell proliferation of A498 cells and 786O cells with indicated treatment. (**D, E**) Transwell assay showed migration and invasion of A498 and 786O cells with indicated treatment. (**F**) flow cytometry was used to determine the apoptotic rates of A498 cells and 786O cells with indicated treatment. * P<0.05 vs. LV-shCtrl; ** P<0.05 vs. LV-sh-hsa_circ_0085576.

After having validated that YAP1 was a target of miR-498, we then investigated whether hsa_circ_0085576 promoted exerted its role in ccRCC by regulating miR-498/YAP1 axis. As shown in Figure 8A, knockdown of hsa_circ_0085576 decreased YAP1 expression and inhibition of miR-498 or overexpression of YAP1 significantly reversed down-regulated YAP1 caused by hsa_circ_0085576 silencing, in A498 and 786O cells. Afterward, CCK-8 assay showed that knockdown of hsa_circ_0085576 significantly inhibited cell proliferation rate, however, this effect was significantly abrogated by co-transfection with miR-498 inhibitors or YAP1 overexpression plasmids, in A498 (Figure 8B) and 786O (Figure 8C) cells. Subsequent transwell assay showed that the inhibition of miR-498 or overexpression of YAP1 partly impaired the hsa_circ_0085576 silencing mediated inhibition of cell migration, and invasion, in A498 (Figure 8D) and 786O (Figure 8E) cells. Similarly, the enhanced cell apoptosis of both cell lines induced by LV-sh-circ0085567 was partly abolished by co-transfection with miR-498 inhibitors or YAP1 overexpression plasmids (Figure 8F).

## DISCUSSION

Many studies have demonstrated that circRNAs participate in the tumorigenesis of ccRCC [18–20]. However, to date, the functions of circRNAs in ccRCC remain largely unknown. Here, we firstly identified a novel circRNA has-hsa_circ_0085576, which was derived from the host gene ASAP1 (Ankyrin Repeat and PH Domain 1) and was cyclized with the head-to-tail splicing of exon 11 and exon 13. ASAP1 is an ADP-ribosylation factor GTPase-activating protein, which is involved in tumor metastasis [21]. These promoted us to explore the function of hsa_circ_0085576 in ccRCC. Hsa_circ_0085576 was found to be significantly associated with tumor size, clinical stage and metastasis in our study. At a functional level, we found that hsa_circ_0085576 facilitated ccRCC cell proliferation, migration and invasion, and suppressed cell apoptosis. In contrast, knockdown of hsa_circ_0085576 showed the opposite effects on ccRCC cells, *in vitro*. More importantly, we found hsa_circ_0085576 promote cell growth and metastasis, whereas hsa_circ_0085576 silencing significantly suppressed cell growth and metastasis, *in vivo*. These data suggest that hsa_circ_0085576 acts as an oncogene in ccRCC.

CircRNAs are generated by the back splicing to be a covalently closed loop without a 5’-cap or a 3’-poly(A) tail. Multiple factors could regulate the biogenesis of circRNAs from the precursor-mRNA, such as alternative splicing factors [22, 23]. Previous studies approved that the roles of circRNAs within cancer cells are to act as miRNA sponges and regulate the expression and activity of the target genes [24]. And studies also indicated that the cytoplasmic localization of circRNA is closely associated with miRNA sponging [25]. Hsa_circ_0085576 was found located in the cytoplasm of A498 cells. Herein, we further investigated the role of hsa_circ_0085576 in ccRCC by miRNA-mRNA mechanism. In accordance with previous studies [13, 26, 27], we presented the ceRNA axis upon hsa_circ_0085576 through a series of thorough experiments. The bioinformatics analysis identified three miRNAs that might interact with hsa_circ_0085576. However, only miR-498 could bind and be regulated by hsa_circ_0085576 in the present study. Luciferase assays and RIP showed that hsa_circ_0085576 interacted with miR-498, which provided evidence that hsa_circ_0085576 competes with miR-498 in ccRCC cells. MiR-498 was identified as a tumor suppressor in several cancers. Zhang et al. recently identified a regulatory network in cervical cancer whereby BMI-1 expression is reduced by miR-498, which are in turn bound by hsa_circ_0007534 acting as ceRNAs, resulting in the restriction of cervical cancer [28]. Besides, circ-PRMT5 could effectively sponge miR-498 to alleviate its repression on the well-known oncogenic EZH2, thereby facilitating lung cancer progression [29]. However, the role of miR-498 in renal cancer is still unrevealed. The expression levels of miR-498 were decreased and negatively associated with hsa_circ_0085576 expression in ccRCC. In loss-of-function experiments, the effects of hsa_circ_0085576 silencing on cell proliferation, migration, invasion and apoptosis could be reversed by a miR-498 inhibitor. Therefore, hsa_circ_0085576 may exert its physiological functions via sponging miR-498.

Recent researches have pointed to an important role for YAP1 signaling in tumor progression [30]. As the well-characterized downstream transcriptional coactivator of the Hippo pathway, YAP1 has been demonstrated to be highly activated in many tumors. YAP1 can promote cancer cell proliferation, migration and invasion through multiple mechanisms [31]. A recent study demonstrated that circ-104075 stimulated YAP1 expression via absorbing miR-582-3p, therefore to stimulate hepatocellular carcinoma tumorigenesis [32]. In the present study, we found that YAP1 was upregulated and positively associated with hsa_circ_0085576 expression. YAP1 was directly targeted by miR-498 and hsa_circ_0085576 indirectly regulated YAP1 expression via sequence matching with miR-498. Moreover, in ccRCC cells, the effects of hsa_circ_0085576 silencing on YAP1 expression and cell proliferation, migration, invasion and apoptosis could be reversed by a miR-498 inhibitor. indicating that YAP1 promotes ccRCC progression. Therefore, hsa_circ_0085576 may regulate the cell growth and metastasis of ccRCC cells by regulating the miR-498/YAP1 axis.

Our study has certain limitations. First, we identified a novel regulatory role of hsa_circ_0085576/miR-498/YAP1 in ccRCC, However, why hsa_circ_0085576 expressed at high levels in ccRCC still unknown. More studies on alternative splicing during the transcription of hsa_circ_0085576 or its coding gene ASAP1, might be carried out to illustrate the regulatory mechanism. In addition, the diagnostic performance of circRNA has been reported in gastric cancer [33], cervical cancer [34], and even acute kidney injury [35]. Our results showed hsa_circ_0085576 may serve as a prognosis marker. However, due to the lack of normal kidney tissues, the diagnostic performance of hsa_circ_0085576 in ccRCC needs to be further illustrated.

In summary, hsa_circ_0085576 could serve as a predictor for clinical outcomes in patients with ccRCC. Hsa_circ_0085576 plays a critical role in promoting cell growth and metastasis of ccRCC by regulating YAP1 expression via miR-498 sponging. Our findings might provide valuable insights into the development of potential therapeutic targets for ccRCC.

## MATERIALS AND METHODS

### Patient tissue samples

The study was approved by the Ethics Committee of Peking Union Medical College Hospital and each participant signed informed consents before sample collection. Animal experiments were performed in line with the protocols approved by the Institutional Animal Research Committee of Peking Union Medical College Hospital. Seventy-six paired ccRCC tumor tissues and adjacent normal tissues were collected from patients in the Department of Urology (Peking Union Medical College Hospital), with a definite pathological diagnosis by two pathologists independently. Adjacent normal tissues were acquired at least 3cm away from the tumor site. The tissues were stored in liquid nitrogen. After histopathological vetting, 45 samples were assigned to the non-metastasis group and the rest were assigned to the metastasis group, according to the Kidney Cancer, NCCN Clinical Practice Guidelines in Oncology [36]. Clinical information of all patients, including age, gender, clinical stage, Tumor stage, lymphatic metastasis, and distant metastasis, were recorded.

### Cell culture and transfection

Normal kidney epithelial cells (HK2) and renal cancer cell lines (786O, A498, Caki1 and ACHN) and were preserved in our lab, as previously described [8]. Cells were cultured in RPMI medium containing 10% fetal bovine serum (Gibco, Carlsbad, CA, USA), 100 units (U)/ml penicillin, and 100 U/ml streptomycin (Gibco) in a humidified incubator with 5% CO2 at 37 °C. The miRNA mimics and pcDNA.3.1 YAP1 recombinant plasmid vectors and their respective control RNAs were purchased from Ribobio (Guangzhou, China). Lipofectamine 2000 (Invitrogen) was used for transfection based on the manufacturer’s instruction. After 50 nM miRNA and 0.5 μg of YAP1 plasmid were used in a 6-well plate for 24 h’ incubation, transfected cells were collected for protein expression analysis.

### Lentiviral transduction

Hsa_circ_0085576 overexpression and silencing recombinant lentiviral vectors were constructed in the pLVX-hsa_circ_0085576 and LV-sh-hsa_circ_ 00855767 vectors by inserting the hsa_circ_0085576 PCR fragment and shRNA, were constructed by Shanghai Genechem Co, Ltd. (Shanghai, China). Control lentivirus particles LV-shRNA-negative control (LV-sh-Ctrl) and pLVX-Ctrl were served as the control. The interference sequence was listed in Supplementary Table 1. The transfection was conducted according to the transfection instructions of lentivirus (Shanghai Genechem Co, Ltd.). After 24 h incubation, the stably infected cells were selected with 2 μg/mL of puromycin. Cells were sub-cultured every 48 h. Stably transfected cells were collected after medical sieve for four generations.

### CCK-8 assay

Approximately 5,000 A498 and 786O cells were plated into 96-well plates and cultured for 0, 1d, 2d, 3d, 4d, and 5d, followed by treatment with 10 μL CCK-8 solution (Beyotime, Jiangsu, China) for 1 h at 37 °C. The absorbance was measured at 450 nm using a microplate reader.

### Wound healing assay

Cells were seeded in 6-well plates and wounds were created by 200-μL pipette tip when cell confluence reached 80%. After washing with PBS, the cells were treated as indicated for 48 h. The healing wounds were photographed twice at 0 h and 48 h after scratching with a digital camera (Leica DFC300FX).

### Transwell assay

The migratory and invasive abilities of A498 and 786O cells were assessed through transwell chambers (8.0-μm pore size polycarbonate filter). In terms of the invasion assay, the upper chamber pre-coated with Matrigel was supplemented with 1×105 cells, while the lower chamber was replenished with RP1640 medium (500 μl) containing 10% FBS. After 24 h, cells of the upper chamber were wiped by a cotton swab and paraformaldehyde was used to fix the invasive cells that were manually counted under a microscope. As for the migration experiment, cells were put into the top chamber without Matrigel. The experiment was conducted in triplicate with the mean value calculated.

### Flow cytometric analysis

The procedures of cell cycle analysis were carried out following the manufacturer’s instructions of Cell Cycle Assay Kit (ab112116, Abcam). Attached cells were harvested cells and fixed in 70% ice-cold ethanol overnight at 4 °C. Finally, cells were stained with RNase A (10 mg/mL) and Propidium Iodide (50 mg/mL) before analyzed by a flow cytometer (FACSCalibur, Bio-Rad, Hercules, CA, USA). Data were analyzed using CELL Quest 3.0 software.

### Apoptosis assay

Apoptosis assay was performed with an Annexin V-FITC/PI apoptosis detection kit (BD Biosciences, San Jose, CA, USA). The cells were trypsinized and washed twice with PBS after centrifugation. Subsequently, cells were resuspended in binding buffer followed by adjusting to the concentration of 1 × 10^6^/ml. The cells were stained with Annexin V (10 μl) and Propidium Iodide (5 μl) per 1ml cell suspension and incubated for 15min at room temperature in dark. Then 400 μl binding buffer was added. At last, the apoptosis rate was assayed by flow cytometry.

### RNA fluorescence in situ hybridization (FISH)

A probe for the has-hsa_circ_0085576 (5’- CAAA GGCGCAGTTCCTTGGGAT-3’) containing a biotin label was used for RNA FISH analysis. The methods were as described previously by Chen et al. [37].

### Real-time quantitative PCR

Total RNA was extracted from pulmonary tissues or cells by Trizol reagent (Invitrogen). RT-qPCR was performed using the SuperScript VILO cDNA Synthesis Kit (Invitrogen) and Sybr Green PCR mastermix (Applied Biosystems, Foster City, CA, USA). Actin was an internal parameter of circRNA and mRNA, while U6 was as an internal parameter of miRNA. Each assay was performed in triplicate and the 2-ΔΔCt method was used to determine the relative expression of genes. Primers were listed in Supplementary Table 1.

### RNA immunoprecipitation (RIP) assay

RIP assay was carried out using the Magna RIP Kit (Millipore, Billerica, MA, USA) in accordance with the manufacturer’s instructions. Antibodies against argonaute 2 (anti-AGO2) (ab57713, Abcam) and immunoglobulin G were used for the RIP assays. Purified RNAs were extracted and the enrichment of circ-008576 and miR-498 was analyzed by RT-qPCR, as described above.

### Luciferase reporter assay

The mutation of hsa_circ_0085576 and the YAP1 3’UTR was performed by changing the conserved binding sites of miR-498 using a Gene Mutation Kit (Takara). A498 and 786O cells (1×10^4^ cells/well) were cultured in 24-well plates and co-transfected with wild-type (WT) or mutant (Mut) hsa_circ_0085576 and miR-NC or miR-498 using Lipofectamine 2000 (Invitrogen). The cells were also transfected with a Renilla plasmid (Internal control). The luciferase activities were measured with a Dual-Luciferase reporter assay system (Promega) according to the manufacturer’s protocol.

### Protein extraction and western blot

Total protein from A498 and 786O cells was extracted using RIPA Lysing Buffer (Beyotime). Protein was separated by electrophoresis in 12% SDS-PAGE and transferred to PVDF membranes, blocked with 5% milk in Tris-buffered saline with Tween 20, and probed with the primary antibodies against YAP1 (ab52771), LATS1 (ab70562), LATS2 (ab110780), TEAD2 (ab92279), TEAD3 (ab75192) and β-actin antibody (MAB8929, 1:1000; R&D systems). After incubation with peroxidase-conjugated secondary antibodies, bands were visualized with enhanced chemiluminescence (Millipore). The relative band intensities were quantified using a ChemiDoc XRS imaging system.

### RNA sequencing and biological analysis

Total RNA was isolated from cells using Trizol, as described above. The quality of RNA was analyzed using a 2100 Bioanalyzer system (Agilent Technologies). In brief, the purified RNAs were subjected to cDNA synthesis followed by adaptor ligation and enrichment with a low-cycle according to instructions of TruSeq® RNA LT/HT Sample Prep Kit (Illumina, USA). The purified library products were evaluated using the Agilent 2200 TapeStation and Qubit2.0 (Life Technologies, USA) and then subjected to sequencing on HiSeq3000. RNA-seq data were normalized in R v3.2.3 statistical environment (http://www.r-project.org) using DESeq Bioconductor library using a negative binomial distribution [38].

The differentially expressed mRNAs were selected for Gene Ontology (GO, http://www.geneontology.org) and Kyoto Encyclopedia of Genes and Genomes (KEGG, http://www.genome.jp/kegg) pathway analysis. Based on the GO categories, the aberrant mRNAs were classified under different GO terms in terms of their characteristics and the enrichment of the GO terms was calculated. The KEGG database was employed to analyze the aberrant mRNAs and the enrichment of different pathways was calculated. The false discovery rate (FDR) was used to evaluate the significance of the P-value and an FDR<0.05 was recommended.

### Xenograft model and treatment protocol

A total of 20 female BALB/c athymic nude mice (aged 4-6 weeks, weighing 16-20 g, SLAC Animal, Shanghai, China) were housed under pathogen-free conditions and randomly divided into the LV-shCtrl group, the LV-sh-circ0085576 group, the pLVX-Ctrl group and pLVX-circ0085576 group (5 per group). The xenograft model was established by subcutaneous injection of A498/LV-shCtrl, A498/LV-sh-circ0085567 or 786O/pLVX-Ctrl, 786O/pLVX-circ0085567 cells (2 × 10^6^ in 0.2 mL PBS/mouse) on the right back of BALB/C athymic nude mice. The two perpendicular diameters of xenografted tumors were measured each time before compound administration, and the tumor volume was calculated by the formula (a × b^2^)/2 (a is the larger and b is the smaller dimension of the tumor). After 30 days following implantation, mice were all sacrificed, the tumors were removed, weighed and photographed. The lungs were harvested for H&E staining and the area of metastatic foci was quantified by ImageJ and normalized to the total area of lung tissues.

### Statistical analysis

The SPSS statistical software version 19.0 was the main tool for statistical analysis. Overall survival (OS) and disease-free survival (DFS) were estimated by the Kaplan-Meier method. Receiver operating characteristic (ROC) curves were used to determine the cut-off threshold value of hsa_circ_0085576. The experimental data were shown as the mean ± standard deviation (SD). Analysis of variance (ANOVA), Student t-test, Wilcoxon matched-pairs signed rank test, Chi-square test and Spearman’s correlation were responsible for p-values. If P < 0.05, the difference was considered statistically significant.

## Supplementary Material

Supplementary Figure 1

Supplementary Tables
