# Supplementary material for: Hsa_circ_0085576 promotes clear cell renal cell carcinoma tumorigenesis and metastasis through the miR-498/YAP1 axis
Source: Aging (Albany NY). 2020 Jun 15;12(12):11530–49. doi: 10.18632/aging.103300 (PMC7343478; doi:10.18632/aging.103300)
Supplement: Supplementary Figure 1 [file aging-12-103300-s002..pdf]

SUPPLEMENTARY FIGURE

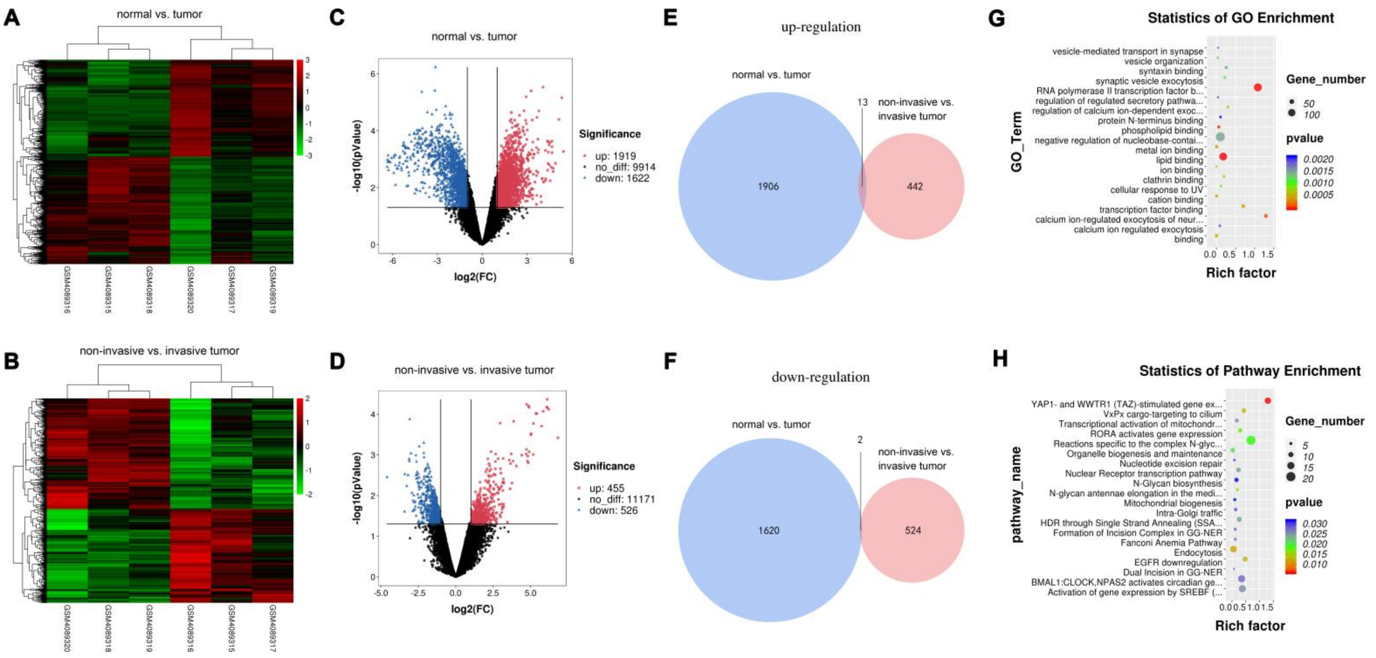

Supplementary Figure 1. Screening of ccRCC-related circRNAs in multiple microarrays. (**A**, **B**) Hierarchical clustering analysis and volcano plots of significantly differentially expressed circRNAs in the tumor or adjacent normal tissues from three patients with ccRCC. (**C**, **D**) Hierarchical clustering analysis and volcano plots of significantly differentially expressed circRNAs in the invasive tumor or non-invasive tumor tissues from three patients with ccRCC. (**E**, **F**) The Venn diagrams demonstrated the up-regulated and down-regulated circRNAs that overlapped during the comparison between tumor and adjacent normal tissues, or between invasive tumor and non-invasive tumor tissues from ccRCC. (**G**, **H**) GO enrichment and pathway analysis for dysregulated circRNAs gene symbols.
