# Supplementary material for: Hsa_circ_0085576 promotes clear cell renal cell carcinoma tumorigenesis and metastasis through the miR-498/YAP1 axis
Source: Aging (Albany NY). 2020 Jun 15;12(12):11530–49. doi: 10.18632/aging.103300 (PMC7343478; doi:10.18632/aging.103300)
Supplement: Supplementary Tables [file aging-12-103300-s001..pdf]

SUPPLEMENTARY TABLES

Supplementary Table 1. The primer sequences of the RNAs used in this study.

Gene	Primer (5'→3')
hsa_circ_0085576-F	CTTTGCAGTGAGCCATGGGA
hsa_circ_0085576-R	GAGTCGTCTCTCCTGTCACG
Liner ASAP1-F	TACCATCAGAGAACAAACACTAATG
Liner ASAP1-R	AAGAACTCTAAGCTGGGTCTGC
SHP2-F	GTCCATGCAGAACGTGAACG
SHP2-R	GCGGGACTGATACTCCTTGA
YAP1-F	TGGGCGGCAACTCCTTCTA
YAP1-R	GCCTCCACGATGAGGGTAAA
TAZ-F	TGGGCTATATCATTGAGTGCAAG
TAZ -R	AAAGACACCGTAGCTGAGGGT
NF2-F	GTCAGCGAGAACAAGGTGC
NF2-R	GATCCGCTCAGCCGTATTCAT
LATS1-F	GAGGCGTGGCAGACTATGC
LATS1-R	CTTGTACTCCGTCAGCGTGA
LATS2-F	GATCACCCGAATGGCTATGAAT
LATS2-R	GGGGTCACAGTTGTCAATGTT
SAV1-F	ATGCAGGATAGCAAGGAGGA
SAV1-R	AAGTGGTCCAACAGCAGCTT
TEAD1-F	CCTGGCTCCTTCAACTGCC
TEAD1-R	GCAAGTAGGTCCAGACAGGT
TEAD2-F	AGACCTCAACCTTAACGAGCA
TEAD2-R	TGTGGAGAGCCTAACTGTTCTT
TEAD3-F	AAGCGACCCAATGGCTTTGT
TEAD3-R	GAGTGTTAGTCTTTCGCAGGG
TEAD4-F	GAGGATAGCTTCACCATCTTGC
TEAD4-R	CCTCAGGAACCGTGTTGGC
MOB1-F	
MOB1-R	
FRMD6-F	TCAACACGACACCGGATAAAC
FRMD6-R	GCCGCGAGCTATCTTTCTTCA
miR-498-F	
miR-498-R	
miR-515-3p-F	AACCAGAGCCTGAGCCAGCTTA
miR-515-3p-R	TGCAGGAAGCGCACGGTCATTT
miR-543-F	GCTGGAGAACGCCGAAGTGCT
miR-543-R	TGGACACGAACGTGTGCACCTC
GAPDH-F	CTCTGCTCCTCCTGTTCGAC
GAPDH-R	GCGCCCAATACGACCAAATC
U6-F	GTGCTCGCTTCGGCAGCACATATAC
U6-R	AAAAATATGGAACGCTTCACGAATTTG

Supplementary Table 2. Differentially expressed 15 circRNAs overlapped between the invasive vs. non-invasive tumor set and the invasive tumor vs. adjacent normal tissue set.

CircRNA ID	Gene Symbol	logFC	Position	Regulation
hsa_circ_0085576	ASAP1	6.3490596	chr8:131104218-131374017	up
hsa_circ_0105034	ERCC4	5.9028925	chr16:14015887-14031715	up
hsa_circ_0004706	PPARA	5.5018349	chr22:46631029-46631406	up
hsa_circ_0009173	SNX5	4.9113692	chr20:17937576-17941969	up
hsa_circ_0006208	NPAT	3.7890772	chr11:108046972-108047817	up
hsa_circ_0040787	KLHDC4	3.6655038	chr16:87764157-87788898	up
hsa_circ_0005117	FOXN2	3.5715788	chr2:48555699-48556261	up
hsa_circ_0007138	PTPRK	3.5039278	chr6:128625812-128643455	up
hsa_circ_0002677	PPM1D	3.4575071	chr17:58733959-58734403	up
hsa_circ_0005048	SYT15	-3.3508076	chr10:46754866-47232156	down
hsa_circ_0102828	EFCAB11	2.989885	chr14:90374859-90398971	up
hsa_circ_0005177	RUFY2	-2.03624	chr10:70153831-70154208	down
hsa_circ_0000619	DENND4A	2.5718625	chr15:65992881-65994228	up
hsa_circ_0085362	TRPS1	-3.04896	chr8:116599227-116635985	down
hsa_circ_0102769	SPATA7	2.1018301	chr14:88897515-88899556	up
